# Micro‐fragmented adipose tissue for the treatment of hip osteoarthritis: A prospective pilot study at 1‐year follow‐up

**DOI:** 10.1002/jeo2.70579

**Published:** 2025-12-28

**Authors:** Marco Zaffagnini, Federico Raggi, Eleonora Carillo, Luca Andriolo, Angelo Boffa, Carola Cavallo, Luca Cattini, Stefano Zaffagnini, Giuseppe Filardo

**Affiliations:** ^1^ Clinica Ortopedica e Traumatologica II IRCCS Istituto Ortopedico Rizzoli Bologna Italy; ^2^ Laboratorio RAMSES, Dipartimento Rizzoli Research and Innovation Technology IRCCS Istituto Ortopedico Rizzoli Bologna Italy; ^3^ Laboratorio di Immunoreumatologia e Rigenerazione Tissutale IRCCS Istituto Ortopedico Rizzoli Bologna Italy; ^4^ Dipartimento di Scienze Biomediche e Neuromotorie (DIBINEM) Università di Bologna Bologna Italy; ^5^ Faculty of Biomedical Sciences Università della Svizzera italiana Lugano Switzerland

**Keywords:** hip, injective, micro‐fragmented adipose tissue, orthobiologics, osteoarthritis

## Abstract

**Purpose:**

Micro‐fragmented adipose tissue (MFAT) has been proposed as a promising option for hip osteoarthritis (OA). The aim of this prospective study was to evaluate clinical outcomes of MFAT injections in patients with hip OA.

**Methods:**

Thirty patients (19 men and 11 women, 55.7 ± 8.2 years) with symptomatic hip OA (Tönnis Grade 1–2) were treated with a single ultrasound‐guided MFAT injection. Patients were evaluated at baseline and 1–3–6–12 months of follow‐up with the Visual Analogue Scale (VAS), Western Ontario and McMaster Universities Arthritis Index (WOMAC), and Harris Hip Score (HHS). Adverse events were also documented. MFAT samples were evaluated for cell analysis and characterization.

**Results:**

No major complications were reported, and only three patients failed. The total WOMAC score significantly improved from baseline (31.2 ± 16.4) only at 1 month (21.0 ± 14.3, *p* = 0.015) and 3 months (19.3 ± 15.8, *p* = 0.012), while VAS and HHS scores showed a significant improvement from baseline to all follow‐ups. The minimal clinically important difference (MCID) for the total WOMAC score was achieved in 56.7% of patients at 6 and 12 months. Better clinical improvement and MCID achievement were observed in mild compared to moderate hip OA. A total of 300.000 cells derived from MFAT produced small colonies from Day 10 (89.9 ± 61.2), increasing by Day 20 (129.9 ± 47.9).

**Conclusions:**

A single ultrasound‐guided intra‐articular MFAT injection represents a safe and promising option for hip OA treatment. However, the clinical benefit was partial, and better outcomes were observed in mild compared to moderate hip OA.

**Level of Evidence:**

Level IV.

**Abbreviations**
BMI: body mass index
CFU‐F: colony‐forming unit‐fibroblast
FBS: foetal bovine serum
GLM: general linear model
HHS: Harris Hip Score
MCID: minimal clinically important difference
MFAT: micro‐fragmented adipose tissue
MR: magnetic resonance
MSCs: mesenchymal stromal cells
OA: osteoarthritis
PBS: phosphate‐buffered saline
PRP: platelet‐rich plasma
RCT: randomized clinical trial
SVF: stromal vascular fraction
THA: total hip arthroplasty
VAS: Visual Analogue Scale
WOMAC: Western Ontario and McMaster Universities Arthritis Index

## INTRODUCTION

Hip osteoarthritis (OA) is one of the most common disabling orthopaedic conditions, significantly affecting the ability to perform daily activities [17]. Its prevalence is increasing over time due to the combined effects of ageing, rising obesity rates, and hip injuries related to sports across the global population [7, 25]. Nonsurgical treatments for hip OA, such as physical therapy, oral medications, and intra‐articular injections of steroids, hyaluronic acid, and platelet‐rich plasma (PRP), offer only limited clinical benefits [2, 3, 13]. In fact, these treatments often show unsatisfactory results, with effects diminishing over time and varying among patients [13]. On the other hand, total hip arthroplasty (THA), the ultimate treatment option for hip OA [18], carries risks of failure and the need for revision surgeries, particularly in younger patients [14, 24]. This has spurred research efforts to find new, more effective and less invasive treatments for hip OA to delay or avoid invasive surgery, especially in young and middle‐aged patients with early OA [31].

Recently, mesenchymal stromal cells (MSCs) have been proposed as a promising option for treating hip OA, due to their immunomodulatory, anti‐inflammatory, and paracrine effects [6, 16]. Among the various sources of MSCs, adipose tissue is becoming the preferred option because of the high yield of cells and pericytes that can be obtained compared to other sources [9, 33]. Due to stringent regulations and the high costs associated with isolating and culturing adipose‐derived MSCs, minimally manipulated approaches are gaining interest [11, 12]. These methods can be obtained directly on site in a one‐step procedure with easier collection and handling [28]. Of the available options, micro‐fragmented adipose tissue (MFAT) has the advantage of providing a high number of cells and growth factors without the need for expansion or enzymatic treatment, thus preserving the integrity of both the cells and the tissue microarchitecture [5, 8]. While MFAT injections showed promising results to address patients affected by knee OA, evidence on the use of this cell‐based injectable approach for hip OA treatment is limited [10, 15, 20, 22, 23, 32].

The aim of this prospective study was to evaluate the clinical outcome offered by a single ultrasound‐guided intra‐articular injection of MFAT in young to middle‐aged patients with symptomatic mild to moderate hip OA.

## MATERIALS AND METHODS

Participant screening was conducted at the outpatient clinic of a highly specialized orthopaedic referral centre. Treatment took place between October 2022 and October 2023. Patients were assessed for eligibility based on the following inclusion criteria: males and females aged from 40 to 70 years with unilateral symptomatic hip OA (Visual Analogue Scale [VAS]—for pain from 4 to 8), with radiographic evidence of hip OA (Grades 1–2 according to the Tönnis classification) or magnetic resonance (MR) imaging findings indicative of chondropathy and mild degeneration of the labrum, and ability to sign informed consent. Exclusion criteria included: patients unable to provide informed consent; body mass index (BMI) > 35; those who undergone an injection of another substance in the last 6 months or surgery on the lower limb within the past 12 months; individuals with other hip pathologies (i.e., avascular necrosis of the femoral head, acetabular protrusion, hip deformity resulting from dysplasia or Perthes diseases) or systemic diseases (uncontrolled diabetes or metabolic diseases, rheumatic pathologies, cancer, alcoholics and/or drug‐addicted); patients with a history of extensive surgery of the hip (i.e., periacetabular osteotomy, osteochondroplasty of the femoral head).

A total of 30 consecutive patients with symptomatic hip OA met the inclusion criteria and were enroled in the study. Radiographs (both anteroposterior and lateral views) and magnetic resonance imaging (MRI) were used to evaluate the treated hips at baseline. OA severity was determined based on Tönnis grading of the radiographs. Baseline characteristics, including patient sex, age, BMI, affected side, symptom duration, OA grade according to the Tönnis classification, as well as clinical characteristics of the included patients, are reported in Table 1. Moreover, the onset of symptoms was 15 cases due to daily activities, 7 related to sports activities, 7 of unknown origin, and 1 resulting from a fall. Finally, a total of 10 patients received previous intra‐articular injections of hyaluronic acid, and one patient received an intra‐articular injection of steroids within 6 months prior to the procedure.

**Table 1:** Baseline demographic and clinical characteristics.^a^

Sex, male/female, *n*	19/11
Age, years	55.7 ± 8.2
BMI	25.8 ± 3.0
Side, left/right, *n*	11/19
Tobacco use, yes/no, *n*	3/27
Symptom duration, mean, months	29.5 ± 36.6
Previous hip intervention, yes/no, *n*	11/19
Tönnis OA grade, *n*	
Grade 1	23
Grade 2	7
VAS pain	5.9 ± 1.6
WOMAC total	31.2 ± 16.4
Pain	6.0 ± 3.1
Stiffness	3.1 ± 1.3
Function	22.1 ± 12.8
HHS	68.8 ± 14.9

Abbreviations: BMI, body mass index; HHS, Harris Hip Score; mo, months; *n*, number; OA, osteoarthritis; SD, standard deviation; VAS, Visual Analogue Scale; WOMAC, Western Ontario and McMaster Universities Arthritis Index.

^a^ Data are reported as mean ± SD unless otherwise indicated.

### MFAT procedure

All patients received a single ultrasound‐guided intra‐articular MFAT injection. The procedure was completed in one surgical session in the operating room and performed by two orthopaedic surgeons with established experience in hip pathologies and orthobiologic approaches. Adipose tissue was collected from the subcutaneous abdominal fat as previously described [1]. Prior to fat extraction, the area was injected with a mixture of adrenaline and lidocaine at high dilutions in 500 mL of saline solution, using a disposable 17‐gauge blunt cannula attached to a 60‐mL Luerlock syringe. The fat was then harvested with a 13‐gauge blunt cannula connected to a 20‐mL VacLok syringe for rapid and minimally traumatic suction. The amount of adipose tissue collected varied depending on the quantity and quality of subcutaneous fat available in each patient. The collected fat was immediately processed using the Lipogems® kit (Lipogems International Spa), as described in previous protocols [26]. The resulting MFAT (5 mL) was then transferred to a 10‐mL syringe for patient injection. One syringe containing 1 mL of the harvested processed adipose tissue was sent to the laboratory of the same institute for biological analyses. The injection was performed under ultrasound guidance through an antero‐inferior approach with the patient lying supine and the hip in slight internal rotation, using a 90‐mm, 18‐gauge needle.

After the procedure, patients were instructed to go home with an elastic compression bandage on the harvesting site, to be worn for 2 weeks. The postoperative plan included rest and avoidance of high‐impact physical activities and strenuous work for at least 2 weeks, with the possible use of crutches and progressive weight‐bearing in case of pain. Light activities, such as using an exercise bike or engaging in aquatic therapy, were recommended once the stitches on the harvesting site were removed, with a gradual return to daily and sport activities as tolerated.

### Clinical evaluation

All patients underwent a clinical assessment prior to the injection procedure and at follow‐up visits scheduled at 1, 3, 6, and 12 months. The primary outcome was determined by the change in the Western Ontario and McMaster Universities Arthritis Index (WOMAC) total score from baseline to 6 months of follow‐up. This score includes 24 items across three categories (pain, stiffness, and physical function). Moreover, other scores were used for patient evaluation, including the WOMAC subscales, the Harris Hip Score (HHS), a 10‐item scale assessing four areas (pain, function, deformity, and range of motion), and the VAS for pain. A further evaluation of the clinical effectiveness was performed to assess the number of patients who achieved the minimal clinically important difference (MCID) for the primary outcome (WOMAC total score) at 6 and 12 months of follow‐up (MCID values: 6.6 and 7.7, respectively).

To evaluate the safety of the treatment, any complications or adverse events were monitored and documented at each follow‐up. Mild adverse events were defined as the presence of significant pain or swelling of the treated hip for at least 3 days after the procedure, as reported by patients, while severe adverse events were defined as any events that resulted in death or were life‐threatening and required hospitalization or an intervention to prevent permanent impairment or damage.

Treatment failure was defined as the need for a subsequent surgical or injection procedure due to continued or worsening hip symptoms. For failed patients, the worst clinical evaluation between baseline and the last available follow‐up was considered for the following assessments.

### Cell isolation and characterization

MFAT samples were sent to the laboratory for cell analysis and characterization. All samples were incubated with collagenase type I 0.05% w/v (Sigma‐Aldrich) at 37°C for 1 h to isolate the stromal vascular fraction (SVF) and then filtered through a 100 μm cell strainer (BD Biosciences). SVF was washed with α‐MEM (Sigma‐Aldrich) containing 15% foetal bovine serum (FBS) and seeded in a Petri dish to evaluate its clonogenic potential, and in a T75 flask to examine its phenotypic markers. To evaluate cell clonogenicity, a colony‐forming unit‐fibroblast (CFU‐F) assay was performed. To this end, 3 × 10^5^ mononuclear cells were plated in Petri dishes and cultured in α‐MEM supplemented with 15% FBS for 20 days. Colonies, defined as clusters containing at least 50 cells, were fixed with methanol and stained with a 2% crystal violet solution (Sigma‐Aldrich). For phenotypic characterization, cells derived from the MFAT were seeded in culture flasks at a density of 20,000 cells/cm^2^ and expanded through one passage in α‐MEM supplemented with 15% FBS. The cells were then detached and resuspended in phosphate‐buffered saline (PBS) containing 0.2% sodium azide (Sigma) and 2% FBS. Flow cytometric analysis was then conducted by incubating the cells for 30 minutes at 4°C with the following fluorochrome‐conjugated antibodies: CD31, CD34, CD44, CD73, CD105 (BD Pharmingen); CD14 and CD90 (BioLegend) and CD45 (DAKO). Isotype‐matched controls were used to determine nonspecific antibody binding.

### Statistical analysis

All continuous data were expressed in terms of the mean and the standard deviation of the mean; the categorical data were expressed as frequency and percentages. The Shapiro–Wilk test was performed to test the normality of continuous variables. The Levene test was used to assess the homoscedasticity of the data. The repeated measures general linear model (GLM) with Sidak test for multiple comparisons was performed to assess the differences at different follow‐up times. The Friedman test, followed by the post hoc Wilcoxon pairwise comparison with Bonferroni correction, was used to assess the difference at follow‐up times for ranking scores. The analysis of variance (ANOVA) test was performed to assess the between‐groups differences of continuous, normally distributed and homoscedastic data; the Mann–Whitney nonparametric test was used otherwise. The Exact method calculation was used for nonparametric test in the presence of small samples. The Spearman's rank Correlation was used to assess correlations between numerical scores and continuous data. The Pearson *χ* ^2^ evaluated using the exact test was performed to investigate relationships between grouping variables.

All statistical analysis was performed using SPSS v.19.0 (IBM Corp.).

## RESULTS

**Figure 1:**
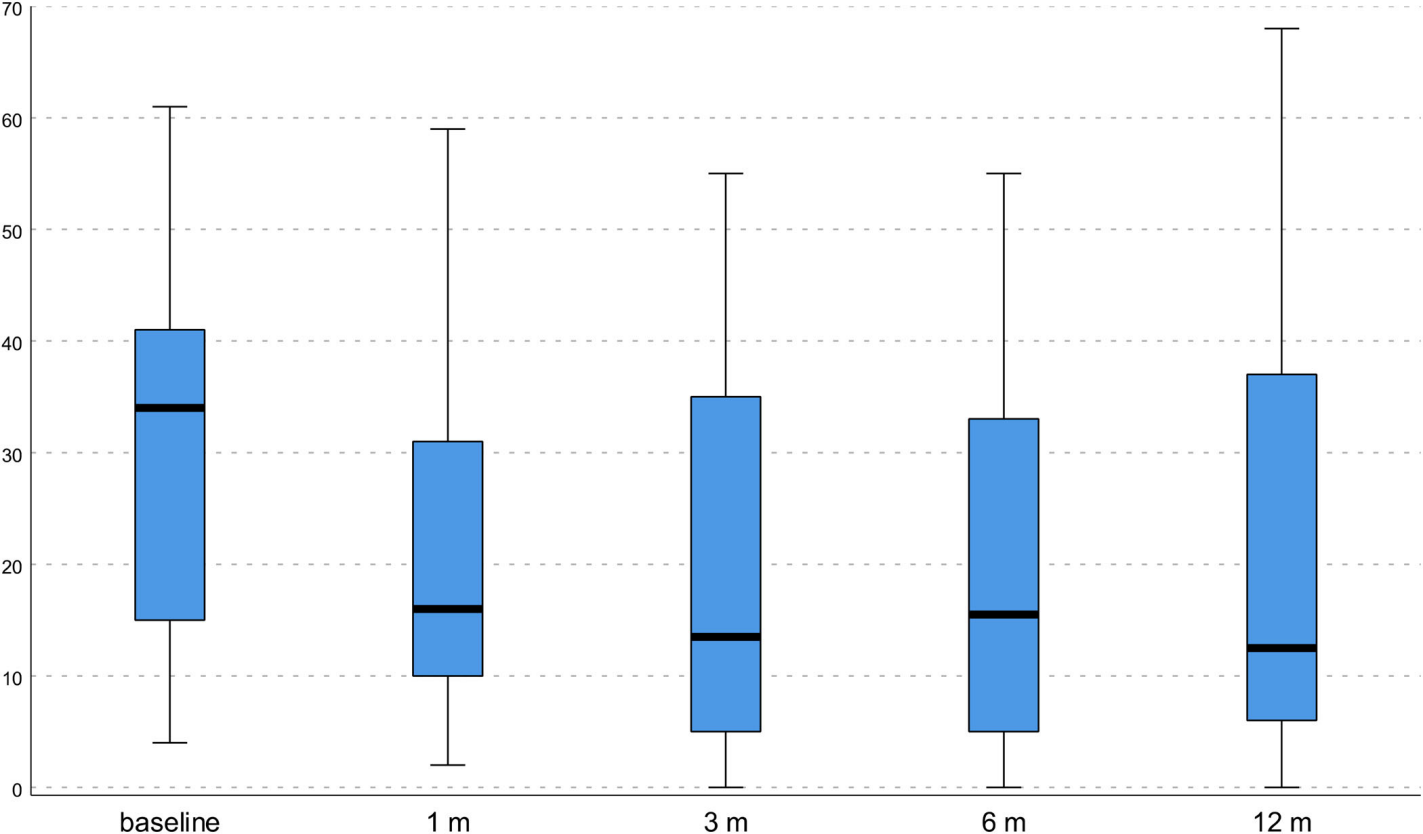
Western Ontario and McMaster Universities Osteoarthritis Index (WOMAC) total score values at baseline and at follow‐up evaluations. The horizontal black line represents the median, the box limit represents the quartiles, and the error bars represent the range.

**Figure 2:**
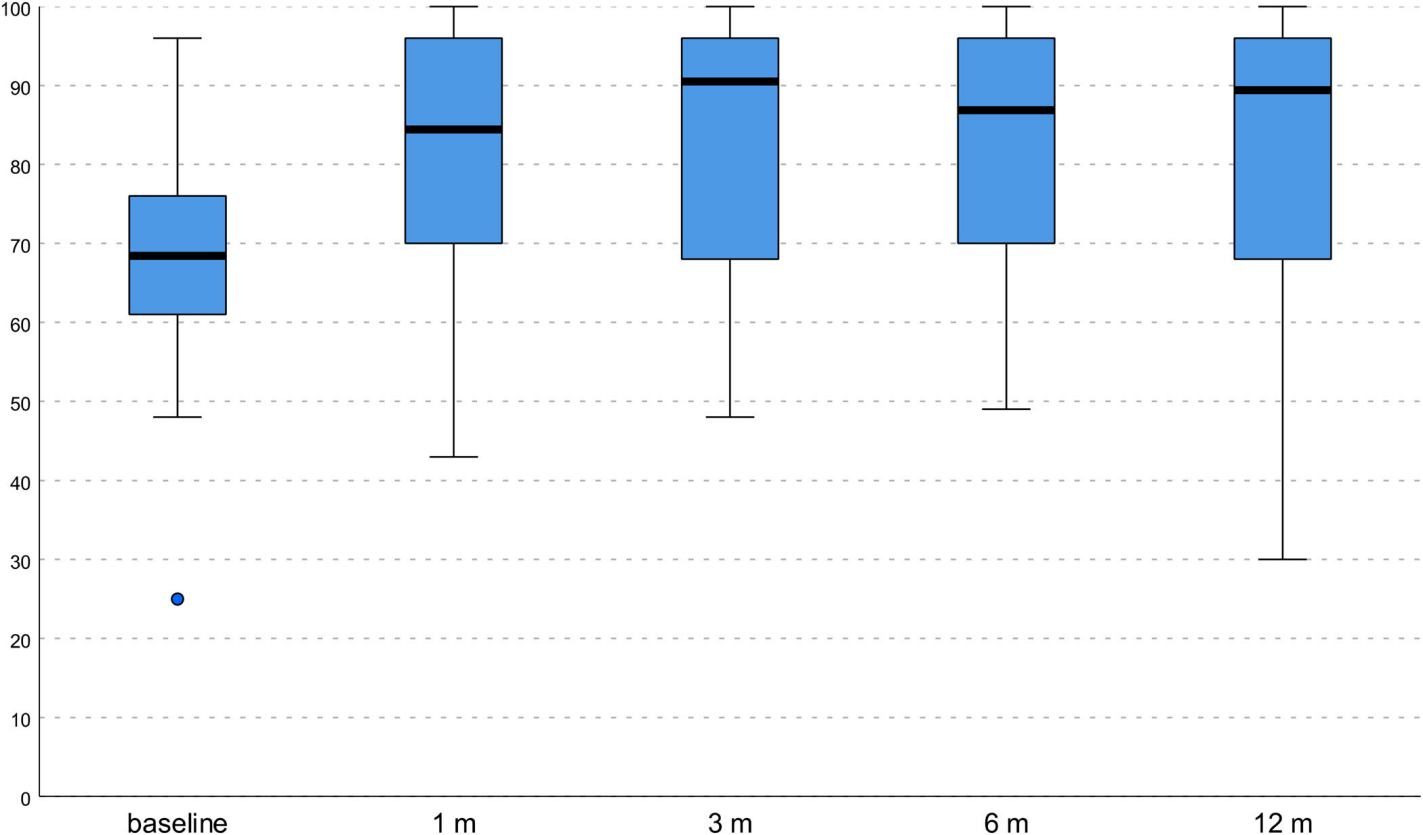
Harris Hip Score (HHS) trend. The horizontal black line represents the median, the box limit represents the quartiles, and the error bars represent the range.

An overall significant improvement in all clinical scores was observed (Table 2). The total WOMAC score showed a statistically significant improvement from 31.2 ± 16.4 to 21.0 ± 14.3 at 1 month (*p* = 0.015), and to 19.3 ± 15.8 at 3 months (*p* = 0.012), while only a tendency (*p* = 0.058) was documented at 6 months (19.9 ± 15.8) (Figure 1), and it did not show any statistically significant improvement at 12 months (20.8 ± 19.3). The HHS showed a statistically significant improvement from 68.8 ± 14.9 to 81.0 ± 14.8 at 1 month (*p* = 0.012), to 82.8 ± 15.5 at 3 months (*p* = 0.002), to 82.9 ± 14.3 at 6 months (*p* = 0.005), and to 82.0 ± 18.4 at 12 months (*p* = 0.024) (Figure 2). Finally, VAS pain showed a statistically significant improvement from 5.9 ± 1.6 to 3.6 ± 1.8 at 1 month (*p* < 0.0005), to 3.7 ± 2.2 at 3 months (*p* < 0.0005), to 3.5 ± 2.3 at 6 months (*p* < 0.0005), and to 4.1 ± 2.4 at 12 months (*p* = 0.002). Detailed values of all clinical scores, including the WOMAC subscales at every follow‐up, are reported in Table 2. The MCID for the WOMAC total score was achieved in 56.7% of patients at 6 months (60.9% for Tönnis 1 patients; 42.9% for Tönnis 2 patients) and in 56.7% of patients at 12 months (65.2% for Tönnis 1 patients; 28.6% for Tönnis 2 patients).

**Table 2:** Improvement in clinical scores from baseline.^a^

Outcome	Baseline	1 month	3 months	6 months	12 months	ANOVA
WOMAC total	31.2 ± 16.4	21.0 ± 14.3*	19.3 ± 15.8*	19.9 ± 15.8	20.8 ± 19.3	*p* = 0.017
WOMAC pain	6.0 ± 3.1	4.4 ± 3.3	3.5 ± 3.1*	3.7 ± 3.3	3.6 ± 3.5	*p* = 0.026
WOMAC stiffness	3.1 ± 1.3	2.3 ± 1.7*	2 ± 1.5*	2.3 ± 1.8	2.1 ± 1.9*	*p* = 0.002
WOMAC function	22.1 ± 12.8	14.3 ± 10.2*	13.8 ± 11.7*	13.8 ± 11.4	14.5 ± 14.0	*p* = 0.029
HHS	68.8 ± 14.9	81.0 ± 14.8*	82.8 ± 15.5*	82.9 ± 14.3*	82.0 ± 18.4*	*p* = 0.006
VAS pain	5.9 ± 1.6	3.7 ± 2.2*	3.5 ± 2.3*	3.5 ± 2.3*	4.1 ± 2.4*	*p* < 0.0005

Abbreviations: ANOVA, analysis of variance; HHS, Harris Hip Score; SD, standard deviation; VAS, Visual Analogue Scale; WOMAC, Western Ontario and McMaster Universities Arthritis Index.

^a^ Data are reported as mean ± SD.

* Statistically significant improvement (*p* < 0.05) from baseline to the follow‐up.

A total of eight mild adverse events occurred after the procedure, including mild or moderate hip pain, joint swelling and/or effusion, and injection site pain lasting for more than three days. All of these adverse events were treatment‐related, self‐limiting and lasted only a few days, with none requiring a specific procedure or hospitalization. Furthermore, two severe adverse events, both unrelated to the treatment, were reported: one patient underwent urgent otolaryngologic surgery at the 1‐month follow‐up, and another patient underwent spine surgery for lumbar stenosis 9 months after treatment. Finally, three patients were considered treatment failures: one patient received a hyaluronic acid injection at 11 months of follow‐up, another patient received both a hyaluronic acid and a steroid injection at 11 months of follow‐up, and one patient underwent THA at 10 months of follow‐up. All failures were patients with Tönnis 2 grade.

**Figure 3:**
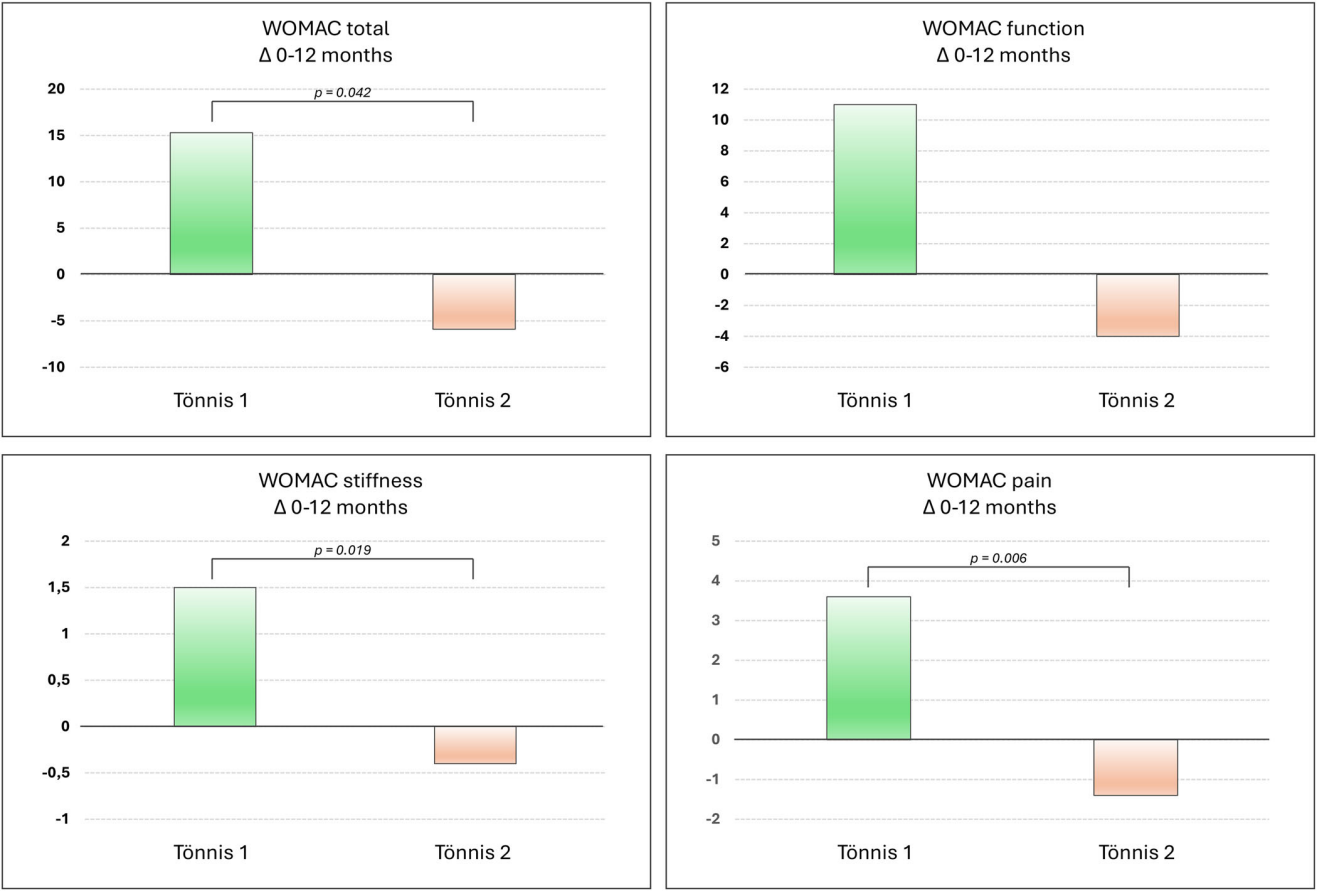
Improvement (*Δ*) from baseline to 12‐month follow‐up of Western Ontario and McMaster Universities Osteoarthritis Index (WOMAC) total score and subscales.

Further analysis was performed to determine the parameters that influenced the clinical outcomes at follow‐up. The severity of OA impacted clinical outcomes, with better clinical improvement from baseline to 12 months in patients with mild OA compared to those with moderate OA in terms of total WOMAC score (15.3 ± 20.8 vs. −5.9 ± 23.3; *p* = 0.042), WOMAC pain (3.6 ± 4.3 vs. −1.4 ± 3.8; *p* = 0.006) and WOMAC stiffness (1.5 ± 1.5 vs. −0.4 ± 1.9; *p* = 0.019) (Figure 3). Conversely, sex, age and previous injections did not significantly influence the post‐injectable clinical outcome.

**Figure 4:**
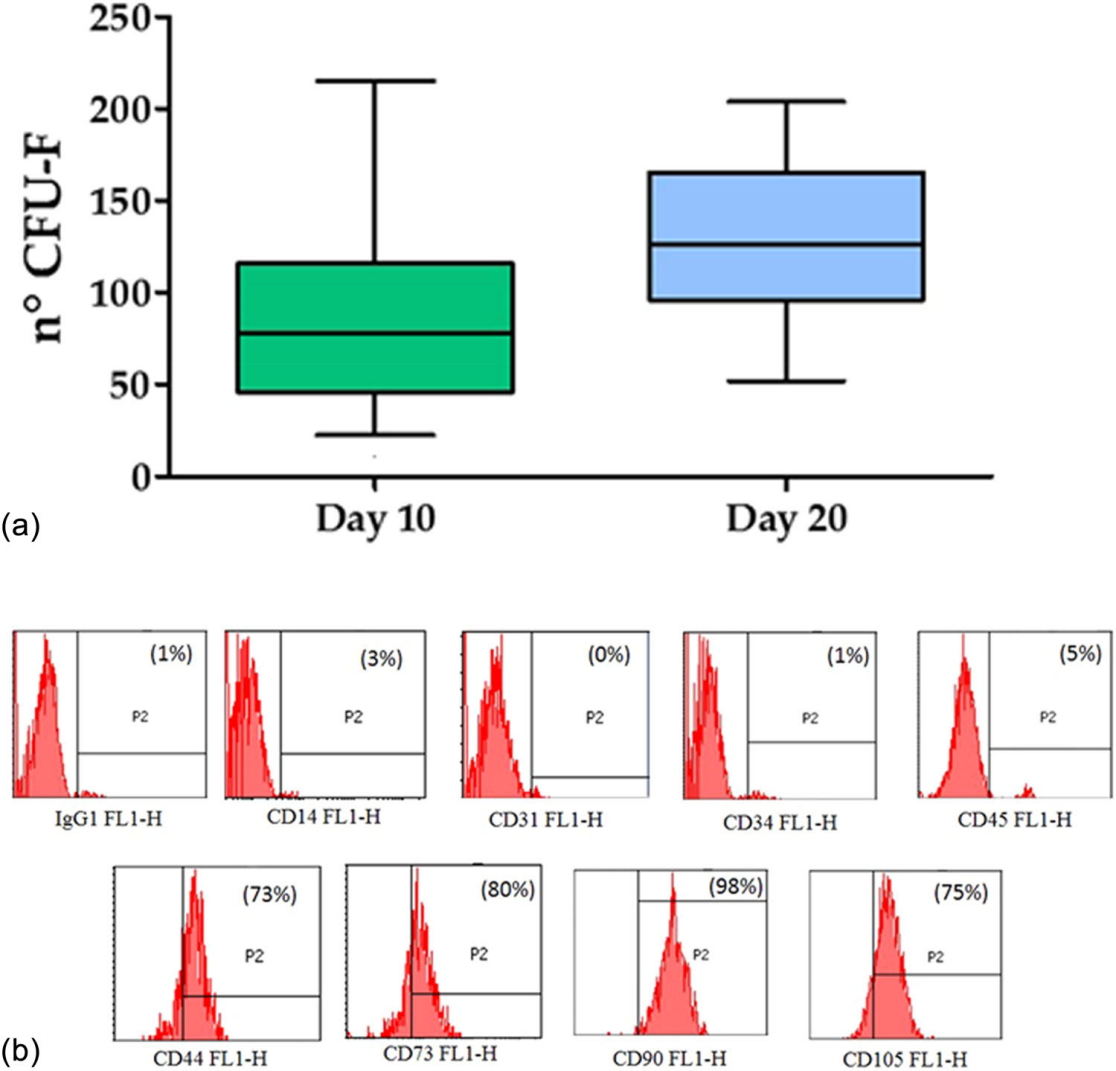
(a) Tukey box plots show the number of CFU‐F at 10 and 20 days. These were obtained by seeding 300.000 mononuclear cells derived from MFAT. (b) FACS analysis of typical haematopoietic and mesenchymal markers in a representative sample of MFAT‐derived cells. CFU‐F, colony‐forming unit‐fibroblast; FACS, fluorescence‐activated cell sorting; MFAT, micro‐fragmented adipose tissue.

The clonogenic potential and the phenotypic characterization were performed on 16 out of the 30 patient samples. Cells derived from MFAT samples showed the capacity to produce a large number of small colonies, starting from day 10 (89.9 ± 61.2) and increasing up to 20 days (129.9 ± 47.9) (Figure 4). No significant differences in the number of colonies were observed between the two days analysed. Flow cytometry analysis showed that all the evaluated samples were positive for typical mesenchymal cell markers such as CD‐44, CD‐73, CD‐90, CD‐105 and negative for haematopoietic markers CD‐14, CD‐31, CD‐34 and CD‐45 (Figure 4).

## DISCUSSION

The main finding of this prospective study is that a single ultrasound‐guided intra‐articular MFAT injection can represent a promising option for the management of patients affected by symptomatic mild to moderate hip OA. This treatment demonstrated safety, with no severe adverse events related to the procedure and a low incidence of mild adverse events and failures. However, its clinical benefit was partial, with only temporary improvement in the primary outcome, and results were influenced by the severity of hip OA, with good results mainly in mild hip OA.

The use of adipose tissue‐derived injectable products is gaining popularity as a possible strategy to treat joints affected by OA. A recent systematic review of preclinical studies on OA models documented evidence of disease‐modifying effects of adipose‐derived cell‐based therapies for the treatment of OA [23]. Some of these preclinical studies evaluated the effect of adipose‐derived product injections for the treatment of hip OA. An intra‐articular injection of adipose‐derived MSCs reduced lameness in eight lame dogs with hip OA [30]. Similarly, a an intra‐articular injection of autologous adipose‐derived SVF and PRP in dogs with hip OA documented greater improvements in function and decrease in pain compared to placebo [27]. Furthermore, an allogenic adipose‐derived MSCs injection in dogs with hip OA reported a greater reduction of pain and lameness compared to placebo [19]. Adipose‐derived MSCs were also compared to PRP in dogs with bilateral hip OA, reporting safety and effectiveness in reducing chronic pain for both treatments, but showing greater improvement compared to PRP treatment [21].

Among the different methods to exploit adipose tissue‐derived MSCs, the MFAT method is emerging as a treatment option to directly harness their biological potential in a single‐step treatment. This approach consists of a straightforward mechanical process that reduces the size of adipose tissue clusters while removing oil and blood residues, without causing significant physical damage to the tissue components or requiring the use of enzymes [26]. In this way, the structural properties and integrity of the microarchitecture of the original tissue are preserved [4] to help maintain the integrity of the adipose ‘niche’ and support the preservation of the MSC microenvironment and their function [12] while providing a viscosupplementation and lubrication effect within the joint compartments [29]. The advantage of preserving the native environment of adipose tissue with MFAT was confirmed by an in vitro analysis: In comparison to an enzymatically processed lipoaspirate, MFAT released a greater quantity of growth factors and cytokines that are involved in tissue repair [29]. MFAT is also rich in microvessels with a high positivity for CD146 and NG2, two pericyte markers, suggesting a higher amount of MSC precursors [6]. In a preclinical study on a rabbit OA model, this approach proved promising, also compared to other procedures requiring higher tissue and cell manipulation [11].

From a clinical perspective, MFAT demonstrated promising results, especially for knee OA [32]. A recent systematic review of the literature included 33 clinical studies focused on the use of MFAT to address knee OA, documenting an overall clinical improvement and a few minor adverse events [28]. On the other hand, clinical evidence on the use of the MFAT approach for hip OA is still limited [31]. Natali et al. conducted an evaluation at a mean follow‐up of nearly 3 years on 55 patients previously treated with a single ultrasound‐guided MFAT injection, documenting clinical improvement in patients with mild to moderate baseline symptoms. However, the authors reported a high failure rate, with 10 patients requiring a new intra‐articular injection and 17 undergoing THA, and they did not assess the progression of improvement over time following MFAT treatment [20]. Similarly, Heidari et al. conducted an observational study in patients with hip OA, comparing a single injection of MFAT (57 patients) with the combined use of MFAT and PRP (90 patients). The authors documented 20 failures, defined as patients who underwent THA and reported clinical improvement in both treatment groups at 12 months of follow‐up, with no significant differences between the two approaches [15]. Despite these preliminary results, there is no prospective evidence on how patients with hip OA respond to MFAT injection treatment over time, and on how the baseline OA severity may influence the clinical outcome.

This study prospectively analysed 30 patients with mild or moderate hip OA who were treated with a single ultrasound‐guided MFAT injection. Clinical assessment was performed at baseline and at 1, 3, 6 and 12 months using different clinical scoring systems to ensure a comprehensive evaluation of each patient. First of all, MFAT injections demonstrated safety, with no major complications related to the procedure, and a low incidence of mild adverse events. Moreover, this approach showed clinical efficacy, reporting benefits in terms of pain, function and symptom relief, with a statistically significant improvement already observed one month after the injection in all the analysed scores. The clinical improvement remained stable up to the last follow‐up for VAS for pain and HHS, while it lost statistical significance after 6 months of follow‐up for the total WOMAC score and its subscales. This benefit was influenced by the OA severity, with better results and a higher MCID achievement observed in patients with early hip OA. On the other side, other demographic characteristics, including age, sex, BMI, months of pain and previous injections, did not influence the clinical outcome. This study reported a failure rate of 10%, with two patients receiving intra‐articular injections for persistence of symptoms and only one patient undergoing THA. This result is of value, considering that this treatment represents a therapeutic option for patients with hip OA who do not respond to previous conservative treatments. In this context, the rate of patients who achieved a clinically significant improvement, exceeding the MCID, was nearly 60% up to 12 months of follow‐up.

This study presents some limitations. First of all, the lack of a control group, inherent to the pilot nature of this study, hindered the possibility to prove the real efficacy of this procedure compared to other strategies, to the natural course of the disease, or to the placebo effect. A further limitation is the limited number of patients, which precluded a significant subanalysis of the factors correlating with the final outcome, although the analysis allowed for the identification of the influence of OA severity on the clinical outcome. The transient improvement in WOMAC scores represents a limitation of this treatment, which yielded better results in patients with mild OA compared with those with moderate OA. Future studies should further investigate this aspect in larger cohorts to confirm the influence of OA severity on the response to MFAT injections. Finally, the study did not include imaging assessments, which could have allowed for the identification of possible effects of MFAT on the progression of OA, and it investigated only the intra‐articular use of MFAT without exploring its intraosseous application. Despite these limitations, the results of this study are relevant, demonstrating that MFAT can be a valuable treatment option in clinical practice for patients with symptomatic hip OA who do not respond to other conservative therapies, especially in early hip OA. The promising results of this study should encourage further high‐level research with a larger patient population, including imaging evaluations and longer follow‐ups, as well as comparisons with placebo and other injectable options, to better understand the therapeutic potential of MFAT injections for the treatment of hip OA.

## CONCLUSIONS

A single ultrasound‐guided intra‐articular MFAT injection can represent a promising option for the management of patients affected by symptomatic hip OA. This treatment demonstrated safety, with no severe adverse events related to the procedure and a low incidence of mild adverse events and failures. However, the clinical benefit was partial, with only 57% of patients achieving the MCID for the total WOMAC score at 6 and 12 months, and better outcomes were observed in patients with mild compared to moderate hip OA.

## AUTHOR CONTRIBUTIONS

**Giuseppe Filardo**: Conceptualization. **Luca Andriolo** and **Federico Raggi**: Methodology. **Marco Zaffagnini, Eleonora Carillo, Angelo Boffa** and **Carola Cavallo:** Data curation. **Marco Zaffagnini, Eleonora Carillo** and **Luca Cattini**: Writing—original draft preparation. **Angelo Boffa, Giuseppe Filardo and Luca Andriolo**: Writing—review and editing. **Giuseppe Filardo** and **Stefano Zaffagnini**: Supervision. All authors have read and agreed to the published version of the manuscript.

## CONFLICT OF INTEREST STATEMENT

S.Z. has received institutional support from Fidia Farmaceutici, Cartiheal, IGEA Clinical Biophysics, Biomet and Kensey Nash; grant support from I+ and royalties from Springer. The funders had no role in the design of the study, the collection, analysis or interpretation of data, the writing of the manuscript or the decision to publish the results. The remaining authors declare no conflict of interest.

## ETHICS STATEMENT

This prospective pilot study was approved by the Hospital Ethics Committee and the Internal Review Board of the IRCCS Istituto Ortopedico Rizzoli, Bologna, Italy (Prot. N. 297/2022/Sper/IOR). The trial was registered at clinicaltrials.gov (registration number NCT05465096), and informed consent was obtained from all participants before enrolment.

## Supporting information

Supplementary materials.

## Data Availability

The data sets generated during and/or analysed during the current study are available from the corresponding author on reasonable request.
