# Supplementary material for: Micro‐fragmented adipose tissue for the treatment of hip osteoarthritis: A prospective pilot study at 1‐year follow‐up
Source: J Exp Orthop. 2025 Dec 28;12(4):e70579. doi: 10.1002/jeo2.70579 (PMC12745173; doi:10.1002/jeo2.70579)
Supplement: Supplementary file 1 — Supplementary materials. [file JEO2-12-e70579-s001.docx]

| Scores | Mean | Standard Deviation | 95% Confidence Intervals | |
| --- | --- | --- | --- | --- |
|  |  |  | Lower Limit | Upper Limit |
| WOMAC Pain Baseline | 6,0 | 3,1 | 4,9 | 7,1 |
| WOMAC Pain 1 Month | 4,4 | 3,3 | 3,2 | 5,6 |
| WOMAC Pain 3 Months | 3,5 | 3,1 | 2,4 | 4,7 |
| WOMAC Pain 6 Months | 3,7 | 3,3 | 2,5 | 4,9 |
| WOMAC Pain 12 Months | 3,6 | 3,5 | 2,3 | 4,9 |
| WOMAC Function Baseline | 22,0 | 12,8 | 17,3 | 26,8 |
| WOMAC Function 1 Month | 14,3 | 10,2 | 10,4 | 18,1 |
| WOMAC Function 3 Months | 13,8 | 11,7 | 9,4 | 18,2 |
| WOMAC Function 6 Months | 13,8 | 11,4 | 9,6 | 18,1 |
| WOMAC Function 12 Months | 14,5 | 14,0 | 9,3 | 19,8 |
| WOMAC Stiffness Baseline | 3,1 | 1,3 | 2,7 | 3,6 |
| WOMAC Stiffness 1 Month | 2,3 | 1,7 | 1,7 | 2,9 |
| WOMAC Stiffness 3 Months | 2,0 | 1,5 | 1,4 | 2,6 |
| WOMAC Stiffness 6 Months | 2,3 | 1,8 | 1,6 | 2,9 |
| WOMAC Stiffness 12 Months | 2,1 | 1,9 | 1,4 | 2,8 |
| WOMAC Total Baseline | 31,2 | 16,4 | 25,0 | 37,3 |
| WOMAC Total 1 Month | 21,0 | 14,3 | 15,6 | 26,3 |
| WOMAC Total 3 Months | 19,3 | 15,8 | 13,4 | 25,2 |
| WOMAC Total 6 Months | 19,9 | 15,8 | 14,0 | 25,8 |
| WOMAC Total 12 Months | 20,8 | 19,3 | 13,6 | 28,0 |
| HHS Baseline | 68,8 | 14,9 | 63,2 | 74,3 |
| HHS 1 Month | 81,0 | 14,8 | 75,5 | 86,6 |
| HHS 3 Months | 82,8 | 15,5 | 77,1 | 88,6 |
| HHS 6 Months | 82,9 | 14,3 | 77,5 | 88,2 |
| HHS 12 Months | 82,0 | 18,4 | 75,1 | 88,8 |
| VAS Baseline | 5,9 | 1,6 | 5,3 | 6,5 |
| VAS 1 Month | 3,6 | 1,8 | 2,9 | 4,3 |
| VAS 3 Months | 3,7 | 2,2 | 2,8 | 4,5 |
| VAS 6 Months | 3,5 | 2,3 | 2,7 | 4,3 |
| VAS 12 Months | 4,1 | 2,4 | 3,2 | 5,0 |
